# Partial regression of foveoschisis following vitamin B6 supplementary therapy for gyrate atrophy in a Chinese girl

**DOI:** 10.1186/s12886-021-01862-1

**Published:** 2021-02-18

**Authors:** Wenxue Guan, Ge Wang, Feng Hu, Xiaoyan Peng

**Affiliations:** Beijing Key Laboratory of Ophthalmology and Visual Science, Beijing Tongren Hospital, Beijing Institute of Ophthalmology, Beijing Tongren Eye Center, Capital Medical University, 100005 Beijing, China

**Keywords:** Gyrate atrophy, Foveoschisis, Vitamin B6, Drug side‐effect

## Abstract

**Background:**

To report a case of genetically confirmed gyrate atrophy (GA) of choroid and retina, who showed partial regression of foveoschisis following vitamin B6 supplementary therapy.

**Case presentation:**

A 6-year-old Chinese girl complained about night blindness and progressive decreased vision in both eyes. Her best corrected visual acuity (BCVA) was 20/63 OD and 20/100 OS. Fundus examination showed bilateral multiple, sharply demarcated, scallop-shaped chorioretinal atrophy areas in the midperipheral and peripheral of the fundus. Spectral domain optical coherence tomography (SD-OCT) showed increased central macular thickness (CMT) with multiple intraretinal cystic spaces in the both eyes. There was no leakage or staining in the macular area in late phase of fluorescein angiography (FA). Blood tests confirmed hyperornithinemia and genetic analysis revealed two heterozygous mutations on ornithine aminotransferase (OAT) gene. Based on clinical presentation and genetic test, the patient was diagnosed as GA of the choroid and retina and further treated with vitamin B6 supplementary for three weeks. Her serum ornithine levels did not change but CMT on SD-OCT declined with partial regression of intraretinal cystic spaces. Then, the patient discontinued the drug because of severe muscle pain, and foveoschisis increased to initial level a month later.

**Conclusions:**

Foveoschisis is a rare complication of GA. Vitamin B6 supplementation may alleviate foveoschisis, but its effort for reducing serum ornithine level might be limited. Potential drug adverse effects should be noted in pediatric patients.

## Background

Gyrate atrophy (GA) of the choroid and retina is a rare autosomal recessive inherited disease (OMIM: 258,870). It is a metabolic disorder due to mutations on the gene encoding vitamin B6 dependent enzyme ornithine aminotransferase (OAT), in which resulted 10 to 20-fold increased level of plasma ornithine [1]. Ophthalmological typical manifestations of GA are scallop-shaped areas of chorioretinal atrophy along with nyctalopia, astigmatism, myopia, early cataract, and significantly diminished or extinguished electroretinogram [2]. Macular involvement has been reported including cystoid macular edema (CME), epimacular membrane, macular hole, choroidal neovascularization and foveoschisis [3, 4]. Foveoschisis is rare in GA with only three cases reported previously [5–7].

Reducing plasma ornithine levels through low-protein or arginine-restricted diet and vitamin B6 supplementation form the basis of GA therapy. Multiple studies have reported that low-protein or arginine-restricted diet may delay the progression of chorioretinal atrophy and visual loss, but most patients fail to follow through [8]. The response to vitamin B6 supplementation varies widely among patients, and only a few patients showed response to this therapy[9]. Moreover, treatment for GA complicated foveoschisis is not available.

Herein, we report a case of genetically confirmed GA who showed partial regression of foveoschisis following vitamin B6 supplementary therapy.

### Case presentation

**Fig. 1:**
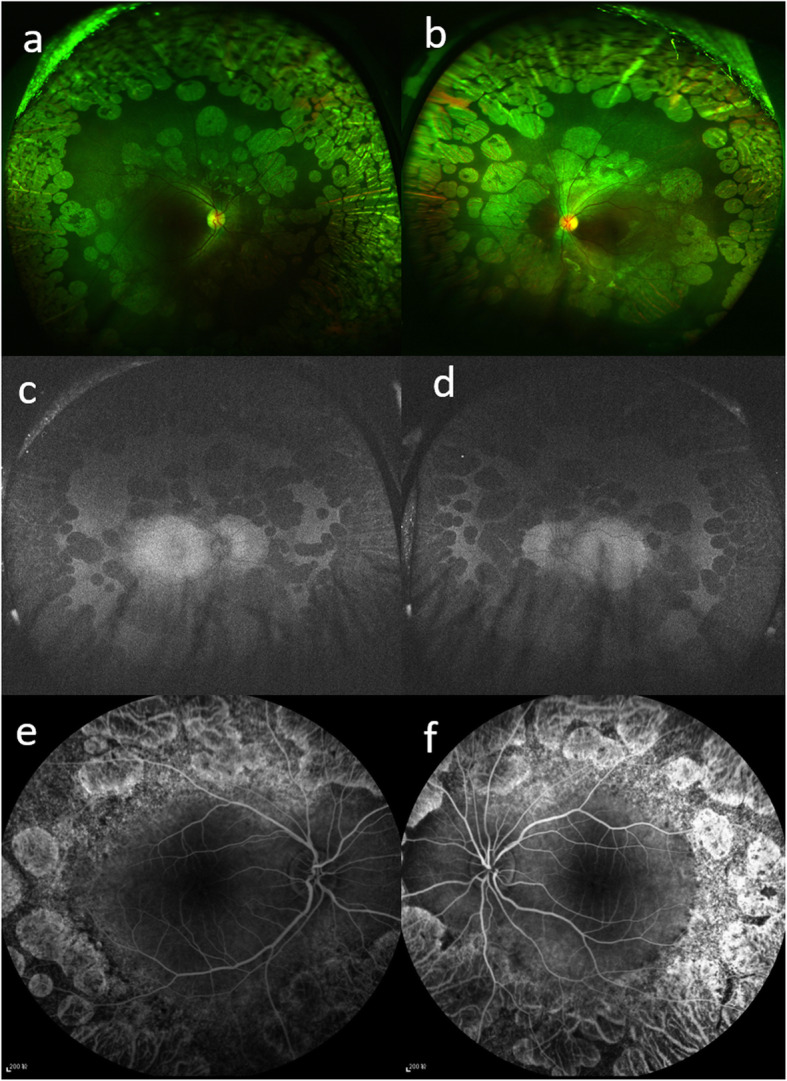
Multiple-mode imaging for a Chinese girl with gyrate atrophy-related foveoschisis **a, b.** Ultrawide field fundus image of both eyes showed multiple, sharply demarcated, scallop-shaped chorioretinal atrophy areas in the midperipheral and peripheral of the fundus. **c, d.** Fundus autofluorescence of both eyes showed decreased autofluorescence in the corresponding chorioretinal atrophy areas. **e, f.** Fluorescein angiography in late phase revealed no leakage or staining in the macular area in both eyes

A 6-year-old Chinese girl is presented to our clinic complaining about night blindness and progressive decreased vision in both eyes for one month. There was no consanguineous marriage and family history of ocular disorders. Best corrected visual acuity (BCVA) was 20/63 in the right eye and 20/100 in the left eye with a refraction error of -7.50 diopter (D)/-2.50D×175° and − 5.00D/-2.75D×180°, respectively. Slit-lamp examination of the anterior segment was unremarkable in both eyes. Dilated Fundus examination showed bilateral multiple, sharply demarcated, scallop-shaped chorioretinal atrophy areas in the midperipheral and peripheral of the fundus (Fig. 1a, b). Fundus autofluorescence showed hypo-autofluorescence in the corresponding area of chorioretinal atrophy (Fig. 1c, d).

**Fig. 2:**
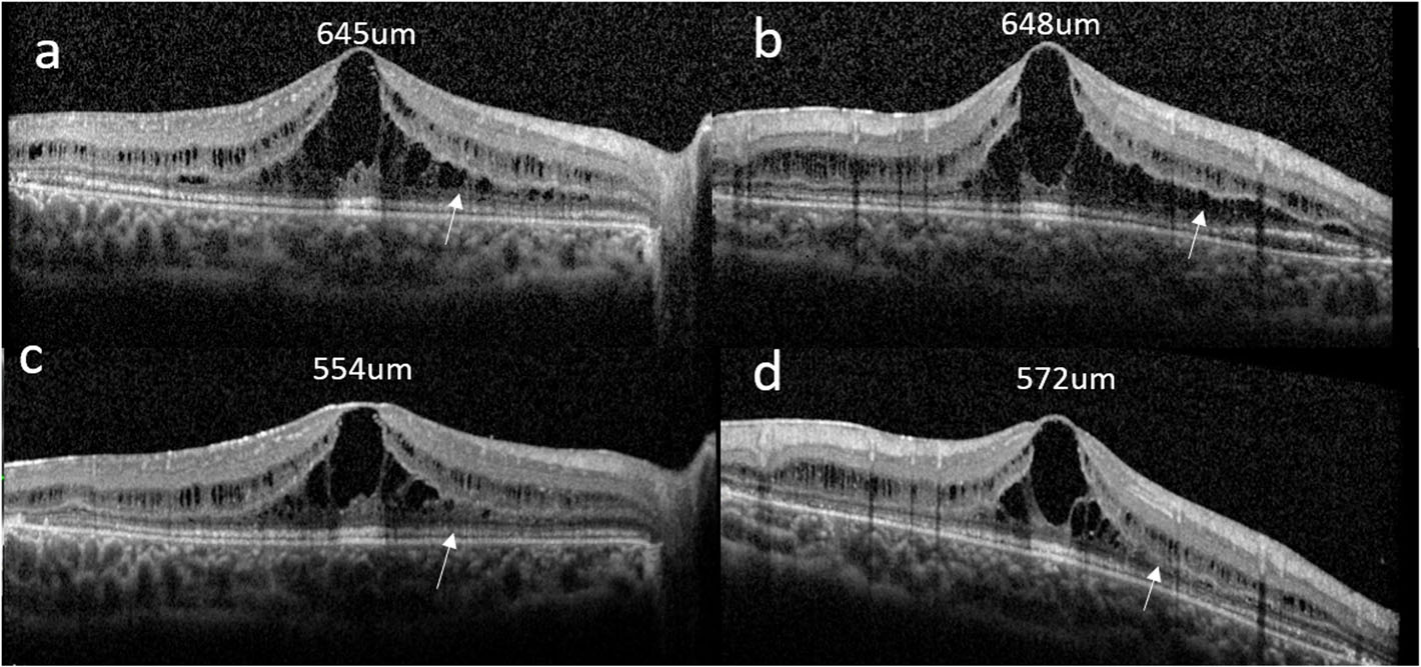
SD-OCT scan before and after vitamin B6 treatment **a, b.** SD-OCT at initial presentation of both eyes showed increased CMT with multiple intraretinal cystic spaces (CMT of 645 µm and 648 µm in the right and left eye, respectively). **c, d.** SD-OCT after vitamin B6 treatment showed reduction of CMT with partial regression of intraretinal cystic spaces (white arrows) (CMT of 554 µm and 572 µm in the right and left eyes, respectively)

Spectral domain optical coherence tomography (SD-OCT) showed increased central macular thickness (CMT) in both eyes (CMT of 645 µm and 648 µm in the right and left eye, respectively), with multiple intraretinal cystic spaces (Fig. 2a, b). There was no leakage or staining in macular area on the late phase of fluorescein angiography (FA) (Fig. 1e, f).

The plasma ornithine level was 1180µmol/L (normal range: 25–115µmol/L), which supported the diagnosis of GA. Whole-exome sequencing in family trios by the Beijing Genomics Institute (Beijing, China) identified two novel mutations in the OAT: (NM_000274.3): c.251 C > T p. (Ser84Phe) and c.648 + 2T > G. Heterozygosity of OAT: c.251 C > T p. (Ser84Phe) was confirmed in the patient’s father and OAT: c.648 + 2T > G was found in the patient’s mother. Vitamin B6 supplementation (dosage of 500 mg/day) was prescribed but low-protein diet is difficult for a 6-year-old patient. The vitamin B6 was reduced to 300 mg/d in the third week due to muscle pain in arms and legs, which was considered as the side effects of vitamin B6. Three weeks after treatment, serum ornithine levels did not decline, despite the patient’s reporting strict adherence to the vitamin B6 supplementation. SD-OCT scans showed reduction of CMT with partial regression of intraretinal cystic spaces (CMT of 554 µm and 572 µm in the right and left eyes, respectively) (Fig. 2c, d). However, the patient discontinued therapy due to severe muscle pain, and CMT increased to its original level a month later. The BCVA in both eyes remained stable during the next five months.

## Discussion

As a rare genetic disorder caused by OAT gene mutations, GA is particularly prevalent in Finland with unknown reasons and it also has been reported in many other countries around the world [10]. This disorder is uncommon in China and more than half of the cases were reported in the western Asian and European countries.

Foveoschisis is commonly related to patients with high myopia, X-linked retinoschisis and Goldmann-Favre syndrome and may also provoke by other conditions including membrane frizzled-related protein gene related ophthalmological syndrome, optic disc pit maculopathy and nicotinic acid maculopathy [11–15]. The formation of foveoschisis is multifactorial which might be related to abnormal vitreoretinal traction, structural instability of the retina caused by nonfunctional proteins, deficiency of essential components by genetic defects or the toxicity of material accumulation. In GA, the high expression of OAT gene in the retinal pigment epithelium (RPE) results in its early impairment and then leads to choriocapillaris and photoreceptors atrophy [16, 17]. Although macula and central vision are typically involved later, foveoschisis may occur early in pediatric group with central vision decrease with unclear pathogenesis [5]. Tekin et al. described a case of foveoschisis associated with GA in low myopia without vitreoretinal traction or optic pits and hypothesized that the foveoschisis might have been triggered by GA [6].

The current literature on the treatment of foveoschisis in GA is scarce. Zhioua Braham et al. reported a 26-year-old female affected by GA with foveoschisis which showed poor response to arginine-restricted diet and vitamin B6 supplementation (300 mg/day) treatment for 6 months [7]. For our patient, foveoschisis showed partial regression after 3 weeks’ high-dose vitamin B6 treatment without an arginine-restricted diet, but the plasma ornithine levels and BCVA remained unchanged. Studies by Casalino and Heller showed reversal of CME and visual acuity improvement after low-protein diet and vitamin B6 supplementation (500 mg/d); however, the serum ornithine level did not decrease significantly in these patients [18, 19]. Therefore, we speculate that the effect of treatment for foveoschisis and CME might be independent on serum ornithine level. In an observational study by Kaiser-Kupfer et al., reducing the plasma ornithine below 400–500 µmol/L by arginine-restricted diet level will slow the progression of visual loss [8]. But the visual outcome, in GA with macular involvement, also depends on the integrity of the macular structure. Our patient did not receive an arginine-restricted or low-protein diet, and this cannot be ruled out as a factor affecting the decrease in plasma ornithine levels and the recovery of macular structures. The long-term visual outcomes should be followed up.

Genetic differences may play an important role in phenotypes and treatment response. We displayed two novel mutation sites on OAT gene that have not been previously reported. In the three formerly reported GA-related foveoschisis, genetic test results were not revealed.

## Conclusions

Foveoschisis is a rare complication of GA, which may occur in the pediatric patients and threaten central vision in early stage. Vitamin B6 supplementation may alleviate GA associated foveoschisis. Potential drug adverse effects should be noted in pediatric patients. Further research is necessary for investigating the pathophysiological mechanism of GA-related foveoschisis.

## Data Availability

All data supporting our findings are contained within the manuscript.

## Abbreviations

BCVA: Best corrected visual acuity
SD-OCT: Spectral domain optical coherence tomography
CME: Cystoid macular edema
CMT: Central macular thickness
D: Diopter
FA: Fluorescein angiogram
GA: Gyrate atrophy
OAT: Ornithine aminotransferase
RPE: Retinal pigment epithelium
